# MONOPOL - A traveling-wave magnetic neutron spin resonator for tailoring polarized neutron beams

**DOI:** 10.1038/s41598-020-62612-9

**Published:** 2020-04-02

**Authors:** Erwin Jericha, Christoph Gösselsberger, Hartmut Abele, Stefan Baumgartner, Bernhard Maximilian Berger, Peter Geltenbort, Masahiro Hino, Tatsuro Oda, Robert Raab, Gerald Badurek

**Affiliations:** 1 TU Wien, Atominstitut, Wien 1020 Austria; 2 Institut Laue-Langevin, Grenoble, 38042 France; 3 Kyoto University, Institute for Integrated Radiation and Nuclear Science, Kumatori, Osaka 590-0494 Japan

**Keywords:** Applied physics, Nuclear physics, Particle physics, Quantum physics, Techniques and instrumentation

## Abstract

We report on first experimental tests of a neutron magnetic spin resonator at a very cold neutron beam port of the high flux reactor at the ILL Grenoble. When placed between two supermirror neutron polarizers and operated in a pulsed traveling-wave mode it allows to decouple its time- and wavelength-resolution and can therefore be used simultaneously as electronically tunable monochromator and fast beam chopper. As a first ‘real’ scientific application we intend its implementation in the PERC (p roton and e lectron r adiation c hannel) project related to high-precision experiments in neutron beta decay.

## Introduction

Recently, a remarkable amount of experimental and theoretical work as well as new models have been published with respect to neutron beta decay. The related topics cover the measurement of correlation coefficients^1–3^, the calculations of radiative corrections^4–7^, unitarity of the quark-mixing CKM matrix^7,8^, the probing of New Physics^9–11^, the flavor composition of astrophysical neutrinos^12^, and the so-called *neutron lifetime puzzle* or *neutron decay anomaly*^13^ in the context of Dark Matter^14–21^, baryon number violation^22–24^, or the Quantum Zeno effect^25,26^. In this publication we introduce a neutron resonance system which has been developed for the purpose of an advanced and flexible neutron beam tailoring at the PERC facility^27^ in the context of high-precision neutron beta decay experiments.

More than four decades ago the concept of spatial magnetic spin resonance (SSR) was introduced by Drabkin^28^. This method exploits the fact that upon passage through an undulatory horizontal static magnetic field ***B***_1_ each neutron in its rest frame experiences an oscillating magnetic field with its own specific frequency according to its velocity and the spatial undulation period of the resonator. If this frequency equals the LARMOR frequency, determined by the strength of a homogeneous static ‘*selector*’ field ***B***_0_ which is applied in vertical direction, a resonant spin flip will take place in close analogy to standard NMR. By placing such a spin resonator between a pair of totally reflecting polarizing mirrors, this effect can be used to single out a specific wavelength (with a certain bandwidth) from an initially polychromatic polarized neutron beam. An obvious application of this concept were electrically tunable neutron monochromators^29–32^ for thermal and cold neutron beams. A thorough theoretical treatment of spatial neutron spin resonance is given in^33^. By running the resonator in a pulsed mode a novel inverted-geometry time-of-flight spectrometer for pulsed neutron sources could be realized, which allowed to perform energy transfer scans without changing the scattering angle^34^. A similar arrangement could be used to demonstrate neutron time-of-flight focusing^35^. Applications of the chopped mode of the Drabkin-type spin filter for pulse shaping were also reported in^36,37^.

Supported by numerical simulations, in 2002 we suggested a novel ‘*traveling-wave*’ mode of operation^38^ aiming to decouple the wavelength resolution of such a spin resonator from its time resolution. Since the wavelength resolution is inherently inversely proportional to the number of spatial field periods, the shortest achievable neutron pulse width is governed by the total length of the resonator if just the current generating the transverse oscillatory field is chopped. If, on the other hand, the magnetic field in each half-period of the resonator can be turned on and off at any moment, independently of what happens in the other resonator regions, the achievable time resolution will no longer be limited by the total length of the resonator but only by that of one half-period, without any deterioration of the achievable wavelength resolution. An appealing feature of this novel concept is that in principle both of these resolutions can be changed not only separately but also almost instantaneously by purely electronic means, provided an appropriate, inevitably rather complex circuitry is used for this purpose.

In recent years we have developed several prototypes of neutron spin resonators with independent elements, able to be operated in such a traveling-wave mode. The first experimental tests at the 250 kW low flux TRIGA research reactor of our university in Vienna could be performed only with a dichromatic beam of thermal neutrons delivered from 1^*st*^ and 2^*nd*^ order (002)-reflection of a highly oriented pyrolytic graphite (HOPG) monochromator. Nevertheless these experiments allowed us to improve the resonator performance step-by-step from one prototype to the next and to demonstrate that our proposed concept is indeed useful for flexible neutron beam property tailoring. The most advanced of these prototypes, named MONOPOL 3.1, was designed and optimized for installation at the PF2-VCN beam line at the high-flux reactor of the ILL Grenoble, where it could be tested for the first time with a ‘*white*’ neutron beam, in this specific case of very cold neutrons. Since the basics of our traveling-wave concept of spatial spin resonance have already been published^39–42^, in what follows we will focus on the description of the essential constructional features of this resonator prototype and on the results of the first experiments. Details of the dedicated electronics which we have developed to run MONOPOL will be described in a forthcoming paper.

## Basic principle

As mentioned already in the introduction, spatial neutron spin resonance has been well-understood for some time, and is thoroughly discussed in the literature^32–35^. Therefore we just give a very brief summary of its theoretical basics. It is well known that an analytical description of the motion of a magnetic moment within an NMR-like arrangement of a static and a transverse oscillating magnetic field can only be given if the transverse field amplitude *B*_1_ is much smaller than the strength *B*_0_ of the static field and hence the *rotating field approximation* can be applied^43–45^. For sinusoidal oscillations the wavelength dependence of the spinflip probability *W*_↑↓_ of a neutron traversing such a field region of length *L* and period length 2*a* is given as a function of the neutron wavelength *λ*,

1 $${W}_{\uparrow \downarrow }(\lambda )=\frac{{\xi }^{2}}{{(\Delta \lambda /\lambda )}^{2}+{\xi }^{2}}{\sin }^{2}\left(\frac{N\pi \lambda }{{\lambda }_{0}}\sqrt{{(\Delta \lambda /\lambda )}^{2}+{\xi }^{2}}\right),$$

where Δ*λ* = *λ* − *λ*_0_, *ξ* = 2*B*_1_/*πB*_0_, and *N* = *L*/2*a* is the number of resonator periods, while

2 $${\lambda }_{0}=\frac{C}{a{B}_{0}}$$

denotes the *resonance wavelength*. The constant *C* is defined by fundamental constants, only: $C\hat{=}\pi h/|\gamma |m=6.7822\times 1{0}^{-15}{{\rm{T}}{\rm{m}}}^{2}=6.7822\,{\rm{m}}{\rm{T}}\AA {\rm{c}}{\rm{m}}$, with the neutron’s gyromagnetic ratio *γ* = − 1.8325 × 10^8^ s^−1^ T^−1^, Planck’s constant *h*, and the mass of the neutron *m*. At resonance wavelength *λ* = *λ*_0_, i.e. Δ*λ* = 0, the basic harmonic frequency *ω*(*λ*) = *C|γ*|∕*aλ* in the Fourier series expansion of the transverse spatial magnetic field oscillations, as "seen" from the neutrons in their resting frame, equals the LARMOR frequency *ω*_0_ = |*γ*|*B*_0_ that is set by the vertical selector field *B*_0_. If in addition the horizontal field is adjusted to fulfill the amplitude condition

3 $$\frac{{B}_{1}}{{B}_{0}}\cdot N=(2k+1)\frac{\pi }{4}\,,\,k=0,1,2,\ldots $$

we obtain *W*_↑↓_(*λ*_0_) = 1. Under these conditions the neutrons undergo exactly 2*k* + 1 spinflips upon their passage and will leave the resonator with inverted orientation of their polarization. An alternative version of this relation for *k* = 0 is given in Supplementary Equation (S1). The highest wavelength resolution is obtained for this *k* = 0 case. It improves with the number of resonator periods *N* according to the relation

4 $$\frac{\Delta {\lambda }_{1/2}}{{\lambda }_{0}}\simeq \frac{0.8}{N},$$

where Δ*λ*_1∕2_ denotes the full-width at half-maximum (FWHM) of the central peak of the spinflip probability (1).

An illustrative example for the quantities involved for an ideal and a realistic resonator setup is given in Supplementary Fig. S1 for a resonator with *N* = 16 periods and a resonance wavelength *λ*_0_ = 2.6Å.

## Methods

**Figure 1:**
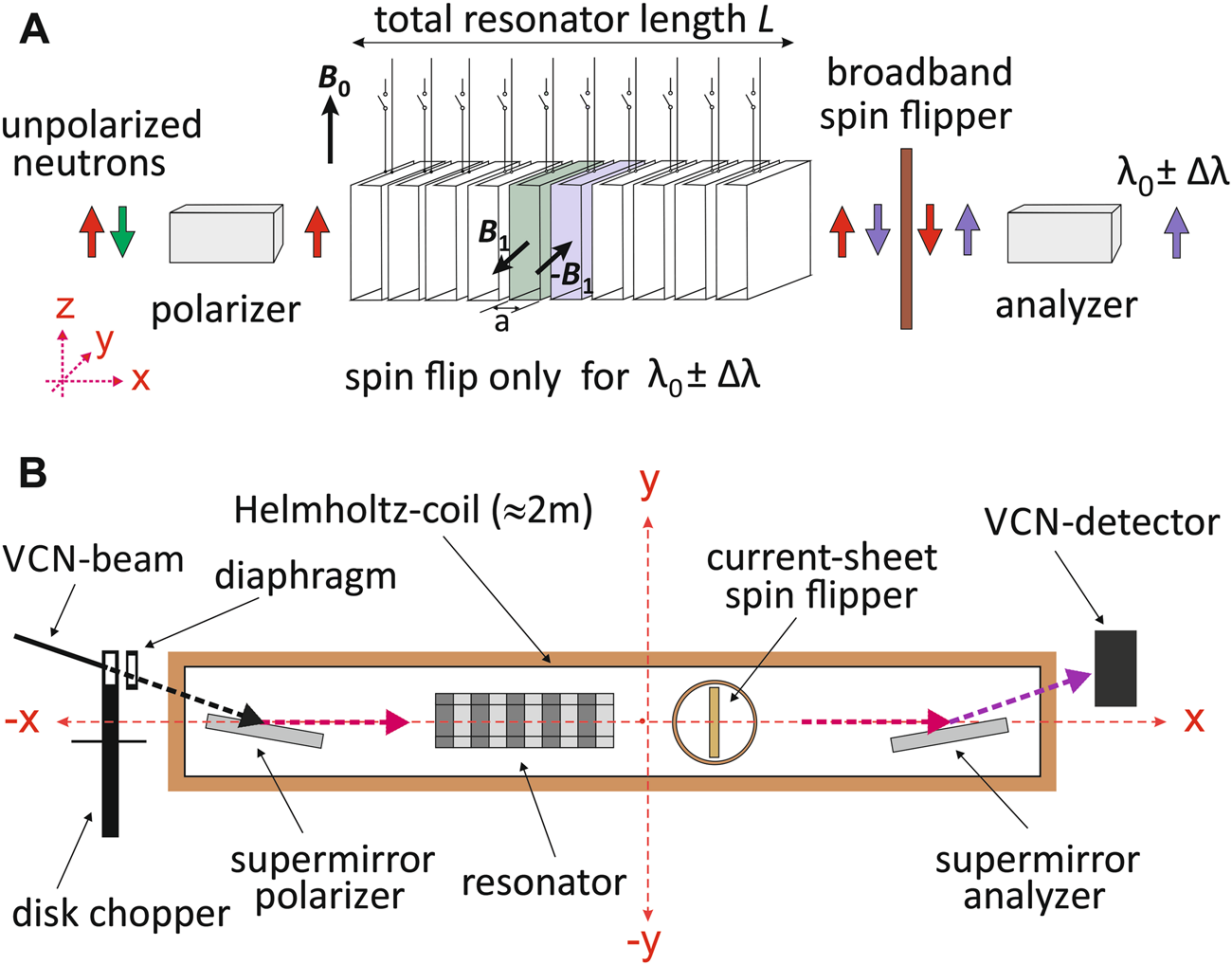
Schematic experimental setup for the spatial magnetic neutron spin resonator MONOPOL at the PF2 VCN beamline. The actual resonator employed consisted of 48 individual single-turn coil elements with a half-period *a* = 11.6 mm. The essential components of the resonator setup shown in the upper image (**A**) were placed in helium atmosphere to reduce absorption and scattering of the VCN in air. The 2.2 m long aluminium container is shown in image (**B**) as large rectangle. A rotating disk chopper and the spatially resolving neutron detector were placed outside the container.

**Figure 2:**
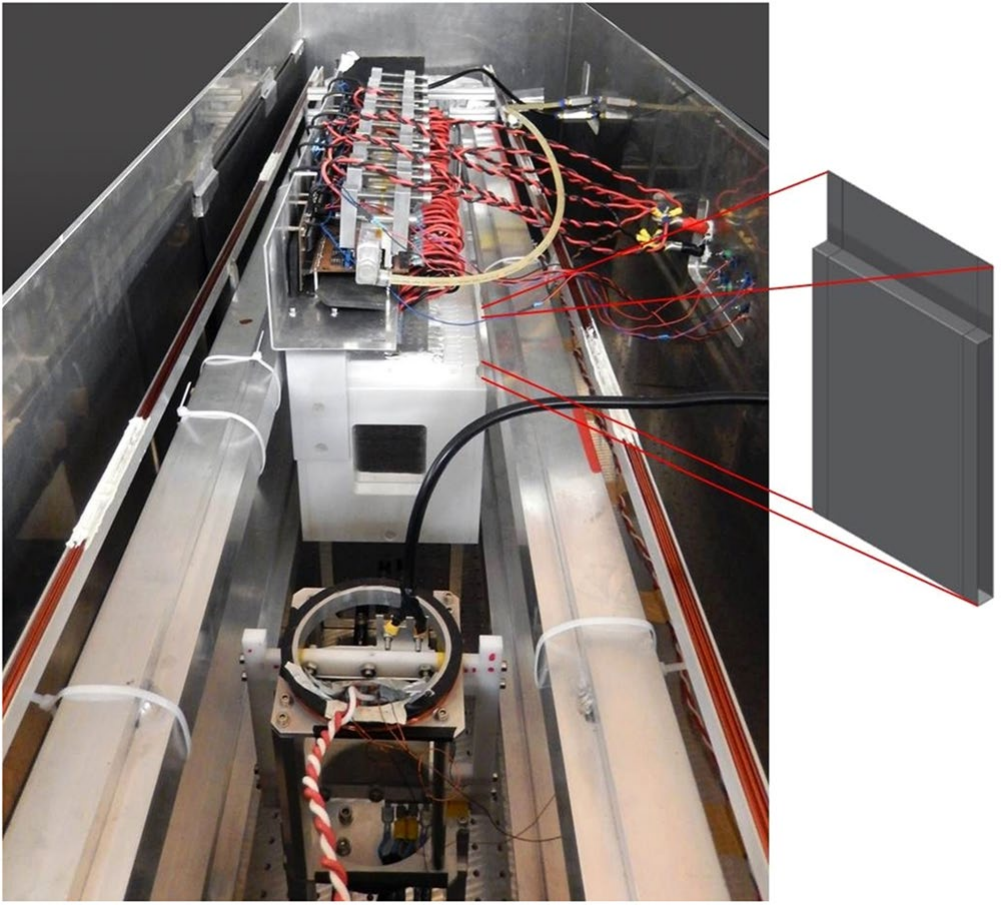
View of the experimental set-up: the resonator with 48 individual elements is shown in the center of the picture together with the individual supply lines, a broad-band current sheet spin flipper for polarisation inversion of the neutron beam is shown in the foreground. The complete experiment is placed in helium atmosphere to reduce neutron scattering and suppress VCN absorption. The sketch of an individual resonator coil, folded from an 0.1 mm thick aluminium foil, is shown at the right side of the photograph.

The experimental setup at the PF2-VCN beam line of the ILL Grenoble to verify the features of our resonator with very cold neutrons is sketched schematically in Fig. 1. The time structure of the VCN beam can be configured either by a single disk chopper or by the resonator itself. The disk chopper used for the experiments has an opening of about 36 mm central width and 19 mm height, corresponding to an angular width of 12.5° or 3.5% of the disk circumference. It may be operated with a frequency in the range 10–30 Hz. Immediately behind the chopper a static boron slit diaphragm with 15 mm width and 20 mm height was installed. The combination of these two openings results in a trapezoidal temporal distribution. At 10 Hz the total opening is about 4.9 ms, the duration between fully ‘closed’ and fully ‘open’ (and vice versa) takes 1.45 ms, and the beam is fully open for about 2 ms. Clearly, for time-of-flight measurements with chopper operation all spectral effects have to be convolved with this distribution. Immediately behind the chopper the beam entered an aluminium box of about 2.2 m length, continuously flooded with helium gas slightly above atmospheric pressure to reduce scattering and absorption in air. The detected neutron intensity increased within the helium atmosphere by a factor 4.7 when compared to air. As indicated in Figs. 1B and 2, downstream the beam trajectory polarizer, resonator, broad-band neutron spin flipper, and analyzer were placed inside this helium box. Polarizer and analyzer were realized as Fe/SiGe-based single-reflection supermirrors mounted within a permanent magnet yoke. Neutrons reflected from the supermirrors were polarized and analyzed, respectively, in positive vertical direction. We have estimated an overall degree of polarization of *P* ≈ 0.95 for the reflected neutron beam. This moderate degree of polarization was sufficient to demonstrate the correct functionality of MONOPOL, but it is clear that for our intended first application (see discussion section) high-performance polarizers with an efficiency close to 100% will be required.

**Figure 3:**
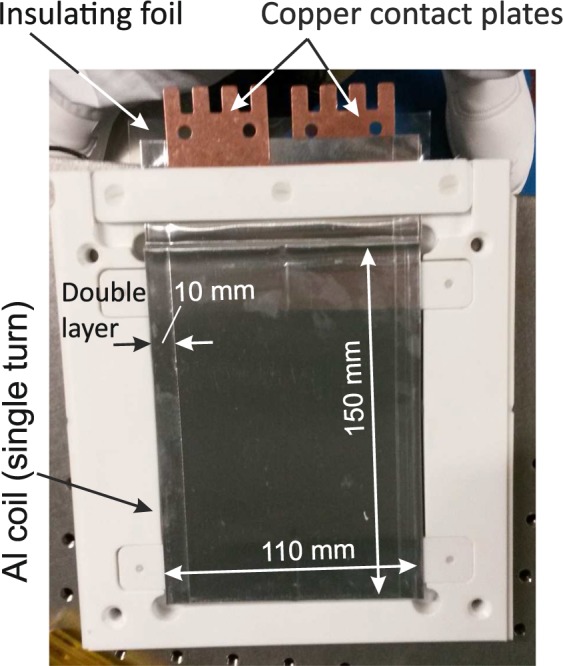
Photograph of a single resonator element consisting of the aluminium coil in the center, a plastic support frame and 2 copper contact plates on top. The double layer on the outer edge of the coil ensures field homogeneity in the center of the coil.

**Figure 4:**
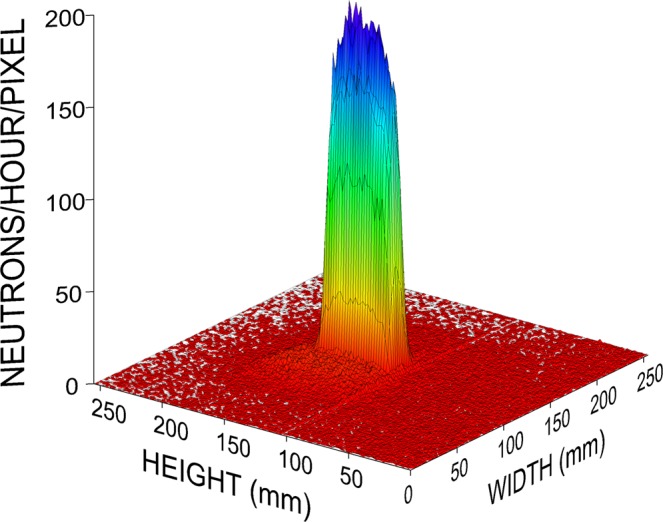
3D-image of the MONOPOL neutron beam in the 2D position sensitive VCN detector. Each of the 128 × 128 detector pixels covers an area of 2 × 2 mm^2^.

A vertical magnetic field, produced by a 2 m long pair of Helmholtz coils which were also placed inside the helium box, both preserved the polarization and acted as the selector field for the resonance wavelength. The resonator consisted of 48 individual elements. Each of these elements has been manufactured from a 0.1 mm aluminium foil and is of prismatic shape with  ≃ 11.0 mm length and 11 × 15 cm^2^ width  ×  height (Fig. 3). The neutrons traverse the aluminium foil twice per resonator element during entry and exit, i.e. 96 times or 9.6 mm in total. The neutron intensity averaged over the incoming VCN spectrum is thereby reduced to 24%. These losses are comparable to the intensity gain obtained by means of the helium atmosphere. Each resonator element is controlled by an individual current source mounted directly above the elements. The current may be switched on and off within a period of about 1 *μ*s. Taking into account the distances between neighboring elements, each element occupies a length of  ≃ 11.6 mm. This distance constitutes the half-period *a* of the resonator. After leaving the resonator the neutrons passed a broad-band current sheet spin flipper consisting of a 0.3 mm thick aluminium sheet, carrying a DC current 100 A, and of compensation coils providing for a local suppression of the vertical guide field. The horizontal magnetic field produced by this current sheet is oriented transverse to the neutron beam trajectory. Before reaching the detector the polarization of the beam was analyzed by the second supermirror which was located immediately at the exit window of the helium box. We used the standard 2D position-sensitive detector BDIM 26 of the VCN beamline. It is a multi-wire proportional chamber (MWPC) with a single ^3^He gas volume and individual read-out of the channels. The detector has a sensitive area of 26 × 26 cm^2^ covered by 128 × 128 channels which corresponds to a position resolution of 2 × 2 mm^2^. For data analysis we defined an 13 × 29 pixel region-of-interest, corresponding to a beam cross-section of 26 × 58 mm^2^ roughly in the center of the detector (Fig. 4). This feature has proved to be very useful for our experiments since we could nicely discriminate neutrons which had passed our setup from neutrons which had been scattered into the detector from somewhere else. It is worth to be mentioned that from the experience we have gained from the experiments described here we later were able to modify the setup as to use a small part of the beam which did not hit the analyzer mirror for monitoring fluctuations of the rector power and variations of the He-concentration along the neutron trajectory. These minor technical modifications we have published meanwhile as dedicated conference proceedings^46^.

## Results

**Figure 5:**
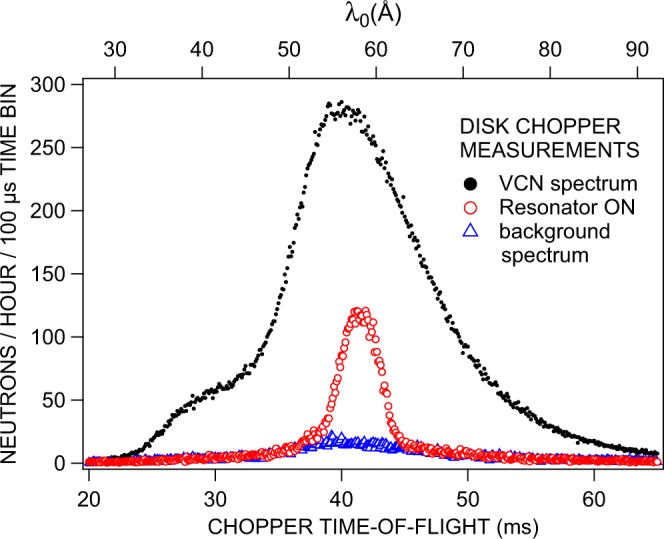
TOF spectrum of the VCN beam as created by a single disk chopper with 10 Hz repetition rate: black symbols (full circles) show the total spectrum when both the resonator and the broad-band spin flipper, which is required to establish a ‘dark field’ configuration, are turned off, red symbols (open circles) illustrate the combined effect of the neutron resonator and the broad-band spin flipper when both are permanently on, blue symbols (open triangles) indicate the neutron background due to non-ideal neutron polarizers when only the current sheet spinflipper is on and the resonator is turned off. The measured TOF spectra are convolutions of the actual spectrum and the chopper transmission function. The timing is started by an optical signal in the chopper electronics and corresponds to a fixed position on the rotating chopper disc. The flight distance between chopper and detector amounts to 2.73 m.

The experiments were performed as time-of-flight measurements. To demonstrate the possibility of spectral beam tailoring, the mechanical chopper in front of the experiment was employed (Fig. 5). Here, the resonator was operated in static mode, i.e. it was permanently switched on when in operation. All displayed TOF-spectra are actually convolutions of the real VCN spectrum with the chopper transmission function. Figure 5 shows the incident VCN spectrum with both resonator and current sheet turned off, the tailored spectrum with both components on, and the background spectrum with only the current sheet on and the resonator turned off. The vertical selector field amounted to *B*_0_ = 101 *μ*T produced by a DC current of 270 mA which results in a resonance wavelength *λ*_0_ ~ 58Å.

**Figure 6:**
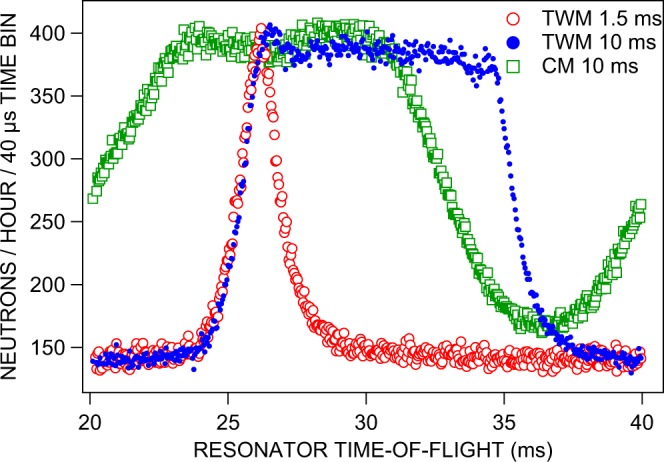
Neutron time-of-flight spectra obtained by operating the resonator either in conventional (CM) or in the novel traveling-wave (TWM) pulsed mode with 50 Hz repetition rate. The typical time scale for VCN is milliseconds and the intensity pattern in the detector repeats every 20 ms. In the conventional mode (open squares) the resonator is switched on as a whole for 10 ms, whereas in the traveling-wave mode (open and full circles) each individual resonator element was switched on (for 1.5 ms and 10 ms, respectively) and off in coincidence with the passing neutron pulse. Obviously, the pulse slopes are considerably steeper in TWM than those achieveable in CM. The timing is started by a software generated signal of the resonator control program. The flight distance between resonator entrance and detector amounts to 1.68 m. The frame for the time axis was chosen in such a way that the depicted values correspond to the actual time-of-flight of the resonant 58 Åneutrons from the resonator entrance to the detector.

**Figure 7:**
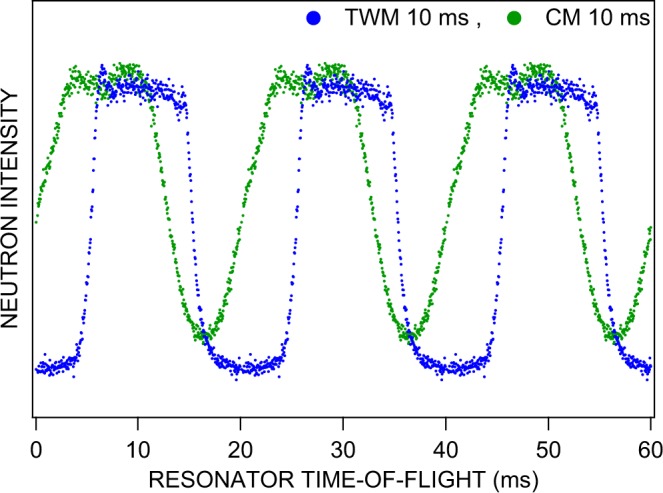
Illustration of the neutron pulses obtained by operating the resonator either in CM or TWM pulsed operation mode with 50 Hz repetition rate and 10 ms switch-on time. The data shown were taken from Fig. 6 and copied to earlier and later times by a shift of  ± 20 ms for each data point. In the experiment the intensity of a large number of pulses was accumulated to obtain the data shown in Fig. 6.

To demonstrate the possibility of temporal beam tailoring, the resonator itself was employed as chopper and pulsed with a frequency of 50 Hz. Corresponding results are presented in Fig. 6 as 3 pulses from different modes of resonator operation. The data shown reproduce the neutron intensity per time bin as recorded by our data acquisition system. The timing was configured with an acquisition time frame of 20 ms duration to match the operating frequency of the resonator. Corresponding time bins with 20 ms distance in time of successive real time frames were added up in a single time bin of the data acquisition to generate time-of-flight spectra of reasonable counting statistics. These sum spectra result from a large number of individual neutron pulses. The temporal sequence of these pulses is shown qualitatively in Fig. 7 which was created by copying the data depicted in Fig. 6 but would have required a much longer measurement time than available in the actual experiment.

In conventional mode (CM) the resonator was switched on and off as a whole. This procedure results in very flat pulse slopes since all those neutrons which are already inside the complete resonator volume during switching times are affected and hence undergo an incomplete spin flip. Note that the time frame settings of our neutron detector resulted in a splitting of the rising slope in CM as shown in Fig. 6. Due to the 20 ms periodicity of the recorded intensity, this slope can be immediately extended to earlier times as it was done in Fig. 7 for the purpose of demonstration. There, the intensity increase between 35 and 40 ms also appears between 15 and 20 ms and gives a complete picture of the pulse structure. Successive pulses were not completely separated for the 10 ms switch-on time and the background level was never reached between the pulses. All neutrons of the 20 ms time frame were at some point affected by the resonator field.

**Figure 8:**
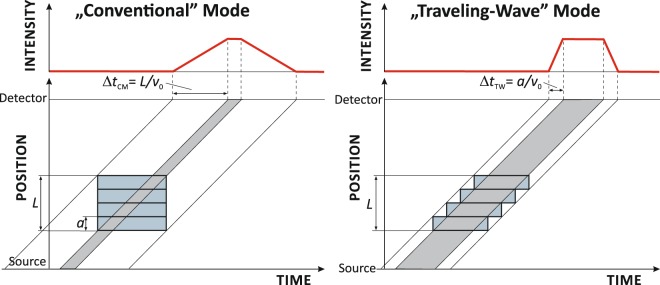
Timing scheme of conventional (CM) versus traveling-wave (TWM) mode of operation.

In traveling-wave mode (TWM), however, each resonator element was switched on when the first neutrons of interest arrived at its entrance. Then this individual element was kept on for a specified amount of time, 1.5 and 10 ms in Fig. 6. The edges of the related temporal distributions are much sharper than in the conventional mode of operation since only neutrons within a single resonator element are affected during switching time. In TWM neutron pulses were well separated for the 10 ms switch-on time as shown in Fig. 7 and the background level was reached before and after the pulse which can clearly be seen in Fig. 6. Eventually, Figure 8 shows schematically the action of the two different modes of operation.

So far we have not taken into account that the Fourier spectrum of the sudden non-sinusoidal oscillations of the transverse resonator field contains not only the fundamental but all odd harmonic frequencies *ω*_*n*_ = (2*n* + 1) *ω*, whose amplitudes *a*_*n*_ ∝ (2*n* + 1)^−1^ decrease with increasing order parameter *n* = 0, 1, 2, 3, …. Therefore spinflip probability maxima will occur also for odd multiples *λ*_*n*_ = (2*n* + 1)*λ*_0_ of the fundamental resonance wavelength *λ*_0_ which is illustrated in the context of Supplementary Fig. S3. This fact can be used to increase the wavelength resolution of the resonator without increasing its number of periods. If by increasing both ${B}_{0}^{{\prime} }$ and ${B}_{1}^{{\prime} }$, belonging to a resonance wavelength ${\lambda }_{0}^{{\prime} }$, by a factor 2*n* + 1 the resonator is tuned to a shorter resonance wavelength *λ*_0_ which is an odd fraction of the original fundamental wavelength, now assuming the role of a higher order resonance ${\lambda }_{0}^{{\prime} }={\lambda }_{n}$, i.e. ${\lambda }_{0}={(2n+1)}^{-1}\ {\lambda }_{0}^{{\prime} }={(2n+1)}^{-1}\ {\lambda }_{n}$. A full polarization inversion is achieved again for ${\lambda }_{0}^{{\prime} }$ but now with a (2*n* + 1)-fold improved resolution

5 $$\frac{\Delta {\lambda }_{1/2}(n)}{{\lambda }_{n}}=\frac{\Delta {\lambda }_{1/2}(0)}{(2n+1){\lambda }_{0}}\simeq \frac{0.8}{N}\cdot \frac{1}{2n+1}\,,\,n=0,1,2,3,\ldots $$

This relation is illustrated in Supplementary Fig. S4 and explained by the fact that the resonance width Δ*λ*_1∕2_(0) at *λ*_0_ is retained at the higher order wavelengths as a kind of phase space transformation.

**Figure 9:**
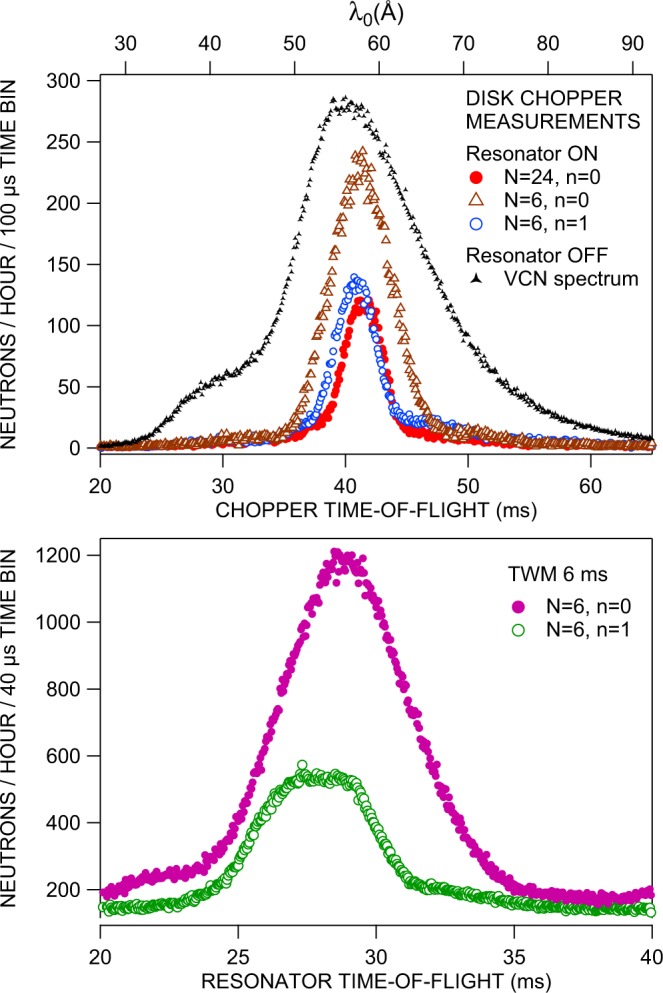
(*Top*) Time-of-flight spectra measured with the mechanical beam chopper and static operation of the MONOPOL resonator for two different numbers *N* of resonator periods and for two different values of the order parameters *n* of the resonance (for details see the text). For comparison purposes the incident VCN spectrum is plotted as well. (*Bottom*) TOF spectra taken for pulsed traveling-wave operation of the resonator with *N* = 6 for both resonance orders (pulse duration: 6 ms, repetition rate: 50 Hz). The measurement principle is equivalent to that presented in Fig. 6 and discussed in Fig. 7.

To demonstrate the dependence of the wavelength resolution both on the number of resonator periods *N* and on the order parameter *n* we have performed a set of measurements, whose results are shown in Fig. 9. First measurements employed the mechanical chopper with the resonator in static mode of operation and are displayed in the upper sub-figure of Fig. 9. Two spectra were taken with the resonator tuned to the same zeroth order (*n* = 0) wavelength *λ*_0_ ≃ 58 *Å*. This implies for both measurements the same selector field *B*_0_ = 101 *μ*T. The transverse resonator field was adjusted according to the amplitude resonance condition of equation (4) to either *B*_1_ = 3.3 *μ*T for *N* = 24 resonator periods, or 13.2 *μ*T for *N* = 6. The two different time-of-flight spectra obtained clearly exhibit the expected loss of resolution with decreasing number of resonator periods. This reduced resolution implies, of course, a corresponding increase of the counts that are accumulated in each detector time channel. A smaller number of resonator periods offers lower resolution, the corresponding curve (open brown triangles in Fig. 9) is broader, and consequently the integrated intensity under the curve is larger. With a higher number of periods the resolution incresases and the curve width decreases which results in an integrated intensity under the curve which is smaller (full red circles in Fig. 9).

Tuning the resonator with *N* = 6 to the *n* = 1 resonance with the fundamental resonance wavelength ${\lambda }_{0}=\frac{1}{3}\times 58=19.33\AA$, whose intensity contribution is negligible in the incident VCN spectrum, the maximum of the measured TOF distribution is located also at 3*λ*_0_ = *λ*_1_ ≃ 58 *Å*. When we interpret the magnetic field values for the *N* = 6, *n* = 0 case above as ${B}_{0}^{{\prime} }=101\ \mu {\rm{T}}$ and ${B}_{1}^{{\prime} }=13.2\ \mu {\rm{T}}$, the required magnetic fields for the *N* = 6, *n* = 1 configuration would have been *B*_0_ = 303 *μ*T and *B*_1_ = 39.6 *μ*T. However, owing to the limited maximum current of our power supply electronics this configuration had to be modified slightly: we have electronically reduced the number of resonator periods by a factor of four from 2*N* = 48 to 2*N* = 12 by respectively merging four neighboring elements of the resonator to one element with fourfold thickness of about 46.5 mm. Now, the required field parameters for this *N* = 6, *n* = 1 configuration were *B*_0_ = 76 *μ*T and *B*_1_ = 9.9 *μ*T, only. The resulting neutron intensity distribution is shown as open blue circles in Fig. 9. Compared to the *N* = 6, *n* = 0 configuration its width is indeed significantly reduced, as expected from equation (5). It has to be added that the evolution of neutron polarization is much more complex in the case of higher order resonances as it is shown in Supplementary Fig. S5. Additionally, the pecularities related to a merging of neighboring resonator elements are discussed in the context of Supplementary Fig. S2.

The small differences between the peak positions of the three wavelength distributions obtained with the three different resonator configurations shown in Fig. 9 can be attributed to shifts of the resonance frequency caused by tiny residual magnetic stray fields of less than 1 *μ*T.

The lower sub-figure of Fig. 9 shows the time-of-flight distributions which were obtained with periodic pulses of 6 ms duration of the *N* = 6 resonator operating in the traveling-wave mode at a repetition rate of 50 Hz for two different order parameters *n*. It becomes evident that only for the 1^*st*^ higher order resonance, *n* = 1, 3*λ*_0_ ≃ 58 *Å*, the wavelength resolution is high enough to yield a pulse plateau (open green circles in Fig. 9), which means that the pulse duration could have been reduced down to about 3 ms without reduction of the peak intensity. It should be noted that for both of these measurements the same procedure as described above with a fourfold resonator half-period *a* = 46.5 mm was applied, which implied that for 0^*th*^ order resonance (*n* = 0) (full pink circles in Fig. 9) both fields were by a factor four (*B*_0_ = 25 *μ*T, *B*_1_ = 3.3 *μ*T) smaller than those in the corresponding static chopper measurement. From the noticeable relative mutual shift of the two time-of-flight distributions by about 2.6% the residual magnetic stray field can be assessed as  ≃ 0.65 *μ*T, which is non negligible at such a small value of *B*_0_.

## Discussion

As a first real application we plan to implement MONOPOL in the PERC ($\underline{\rm{P}}$roton and e lectron r adiation c hannel) facility^40^. PERC will be installed at the MEPHISTO beam line of the high-flux reactor FRM II in Garching near Munich, Germany by an international collaboration of several institutions, Technische Universität München, Universität Heidelberg, Universität Mainz, Stefan Meyer Institute of the Austrian Academy of Sciences, Technische Universität Wien, and the Institut Laue-Langevin in Grenoble^47,48^. PERC is a new user facility for high-precision measurements of angular correlations in the beta decay of free cold neutrons, delivering an intense beam of decay electrons and protons whose spectra and angular distributions will be free of distortions and background about ten times better than hitherto achieved, i.e. at a level of 10^−4^. Likewise important, the phase space density of PERC will exceed that of existing neutron decay spectrometers by several orders of magnitude. It is clearly intended to provide new insights into the Standard Model (SM) of particle physics and fields and beyond.

Neglecting the T-violation in the SM description of particle physics, it requires only three parameters to describe free neutron decay, namely the Fermi coupling constant *G*_*F*_, the quark mixing CKM matrix element *V*_ud_, and the ratio *λ* = *g*_A_/*g*_V_ of axial and vector coupling constants^49^. This means that high-precision measurements of the large number of weak interaction parameters that are accessible in free neutron decay should be appropriate for the search of new physics beyond the Standard Model^50–53^. Thus far the SM predictions and experimental findings agree at the 10^−3^ level and place severe constraints on the beyond the SM mass scale, though the existence of a few significant disagreements may signal the existence of new physics below this scale^54^. An analysis of the sensitivity to deviations of the order of 10^−4^ from the SM is given in^55^ for the neutron lifetime as well as for all experimentally observable asymmetries and energy distributions in the *β*-decay of both unpolarized and polarized neutrons.

To achieve the desired extremely high precision it will be mandatory to install the most advanced available technology at PERC. This will be of particular importance for its pulsed mode of operation with polarized neutrons. The MONOPOL technique has the appealing feature that it requires no mechanical velocity selector and offers a much higher flexibility with respect to the wavelength resolution (typ. Δ*λ*/*λ* ≈ 10%) and the width and repetition rate of the neutron pulses. To realize an overall accuracy level of 10^−4^ it will however be necessary to establish a continuous and stable operation of a highly polarized (≥99.5%) neutron beam for at least about 2 days. Because of this challenging stability criterion spin polarized ^3^He spin filters are out of consideration, whereas high-performance transmission supermirror polarizers^56^ probably should be the optimal choice. Reflection supermirror polarizers are less suited since they would imply different beam trajectories for polarized and unpolarized modes of operation of PERC.

**Figure 10:**
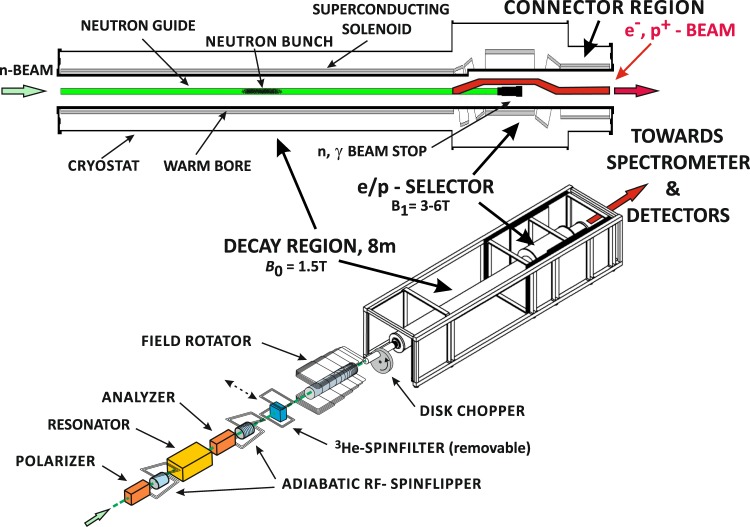
Experimental PERC setup for polarized beam mode of operation with the implementation of the MONOPOL technique. For details see the text and^47,48^.

 Fig. 10 shows the implementation of the MONOPOL technique in the setup of PERC for polarized beam mode of operation. The spin resonator, which can be operated either in static or traveling wave pulsed mode as described in the previous sections, is placed between two high-performance transmission type supermirror polarizers. A broadband radio-frequency spin flipper based upon adiabatic fast passage (AFP) inverts the polarization to establish a ‘dark field’ configuration, so that only neutrons within a small wavelength band around the chosen resonance wavelength are transmitted. A second AFP flipper is used to set the desired spin state of these transmitted neutrons. Notice that with such well-established and widely used type of flippers there is no material in the beam. A removable ^3^He spin filter serves to check its proper function if necessary. Finally the vertical magnetic guide field is rotated adiabatically into the horizontal beam direction, so that the neutron polarization is transferred without any losses into the strong ( ≃ 1.5 T) horizontal magnetic field produced by a superconducting soleneoid within the 8 m long decay region of PERC^27^. The cross-section of the cold neutron guide within that region is 6 × 6 cm^2^. A mechanical rotating slit chopper in front of the decay region, which is standard for conventional pulsed beam operation of PERC, may be used for background minimization provided it is properly phased with the pulses delivered by the MONOPOL arrangement.

During their flight through the decay region a small fraction of the incident neutrons decays. Owing to the expected high flux of the MEPHISTO beamline, the number of decays has been estimated to be of the order of 10^6^ per meter of the decay volume and second. The charged decay products are separated from the neutron beam by a series of high-field (3 to 6 T) superconducting tilted bending and straight filter coils and guided via the connector region towards the detectors^27^. If necessary, further magnetic spectrometers can be installed in front of the detectors, with an example given in^57,58^.

As a final note to our discussion, we did not mention so far that we have tested also the capability of our power supply electronics for Gaussian-shaped amplitude modulation of the transverse field oscillations of the resonator. For the purpose of the study presented here, the characterization of the timing behavior and the wavelength resolution achievable with various configurations, it turns out that such a modulation adds no significantly new information at the given quite high background conditions due to the moderate degree of beam polarization. A resonator with Gaussian-shaped amplitude modulation exhibits a slightly worse wavelength resolution compared to a resonator with uniform magnetic field strength in each resonator element, and therefore a slightly higher transmitted neutron intensity. A short explanation for this behavior is given by the fact that the main contribution to the overall field integral is concentrated in the center of the resonator where the magentic field amplitudes are considerably higher than in the outer parts. This leads to an effectively shorter resonator where the outer regions are configured in such a way that subsidiary maxima, which appear in the oscillatory behavior of equation (1), are damped. Our experimental conditions did not allow us to bring out this effect significantly. However, in the case of high-performance polarizers for thermal and/or cold neutrons amplitude modulation without doubt will be essential.

Currently we are developing an advanced highly flexible and fully computer controlled version of the electronics of MONOPOL with enhanced power to be used also for thermal and cold neutrons. It will be able to deliver individually adjustable bipolar currents to each resonator element, so as to realize - if necessary - a $\sin (x)$/*x*-amplitude modulation for complete suppression of any disturbing subsidiary maxima of the spinflip probability.

Undoubtedly, MONOPOL’s feature to decouple time- and wavelength resolution from each other is increasingly more important with increasing length of the resonator, respectively of the number of its elements. To conclude, we are convinced that the MONOPOL technique will become an indispensable pulse tailoring device to realize ultra-short wavelength-selected sub-pulses of typically 10 to 100 *μ*s duration^42^ at long-pulse neutron sources as the European Spallation Source ESS .

## Supplementary information

Supplementary Information.
