# Supplementary material for: MONOPOL - A traveling-wave magnetic neutron spin resonator for tailoring polarized neutron beams
Source: Sci Rep. 2020 Apr 2;10:5815. doi: 10.1038/s41598-020-62612-9 (PMC7118124; doi:10.1038/s41598-020-62612-9)
Supplement: Supplementary file 1 — Supplementary Information. [file 41598_2020_62612_MOESM1_ESM.pdf]

MONOPOL - A traveling-wave magnetic neutron spin resonator for tailoring polarized neutron beams

Erwin Jericha[1,*], Christoph Gösselsberger[1], Hartmut Abele[1], Stefan Baumgartner[1], Bernhard Maximilian Berger[1], Peter Geltenbort[2], Masahiro Hino[3], Tatsuro Oda[3], Robert Raab[1], and Gerald Badurek[1]

[1]TU Wien, Atominstitut, Wien, 1020, Austria
[2]Institut Laue-Langevin, Grenoble, 38042, France
[3]Kyoto University, Institute for Integrated Radiation and Nuclear Science, Kumatori, Osaka, 590-0494, Japan
[*]erwin.jericha@tuwien.ac.at

ABSTRACT

This document offers supplementary information on magnetic neutron spin resonance with traveling-wave magnetic resonator fields. We present various calculations for the magnetic resonator field, the exit polarization at the end of the resonator and the evolution of the beam polarization inside the resonator for different resonator configurations. Such calculations offer insight into the specific properties of a resonator under real-instrument conditions which allows us to assess its impact on the wavelength spectrum and the degree of polarization for a neutron beam affected by the resonator. These calculations can be performed without any smearing effects induced by neutron optical components and provide us with complementary virtual information that is not accessible by real neutron experiments. Nevertheless, the configurations discussed in this supplementary article are closely connected to the performed experiments which are presented in the *Results* section of the main article.

The magnetic field for the prototype configurations discussed in the following was realistically modeled and calculated using the CST® Studio Suite® software package[1]. Starting with a geometrical representation of the resonator and its elements, a current is sent through the specially shaped conductor material and the resulting magnetic field calculated by finite element calculations. The resulting field configuration is then used to assess the evolution of neutron polarization throughout the resonator for neutrons of varying wavelength. The discussion follows essentially chapter 5 in reference[2].

S1 The resonator in standard configuration

We start with the standard configuration of the resonator which is the equivalent to the original Drabkin resonator[3]. Our first experiments were performed employing this configuration and the corresponding results are shown in Figure 5 and 6 of the main article. The effect of the resonator on the traversing neutrons may be conveniently studied by following the evolution of the neutron polarization throughout the resonator volume. Such calculations allow us to determine the spinflip probability as a function of neutron wavelength for a specific set of resonator parameters. Under ideal resonator conditions the spectral dependence of the spinflip probability will be given by equation (1) which can be verified by our calculations. In addition, our calculations may be performed for more realistic magnetic field configurations which essentially reproduce the conditions of the actual experiments. We find that under such realistic conditions the behavior of the resonator is still described very well by equation (1) for the wavelength range around the fundamental resonance wavelength λ_0 but may exhibit deviations in the spinflip probability of higher order resonance wavelengths. In any case, the best resonator performance is ensured only after a delicate optimization process of the real magnetic field configuration which has to be put into practice up to a certain degree of accuracy for specific instrument requirements. In the case of resonance the flight distance of the neutrons to undergo a single Larmor rotation equals the length of single resonator period. This behavior can be visualized by monitoring the horizontal components P_x and P_y of the neutron polarization vector throughout the complete resonator. In total, the number of Larmor rotations performed equals the number of resonator periods. The quality of resonator parameter optimization may be determined from the magnitude of the rotation remaining after exit from the resonator. In the following, we will illustrate these general statements by an exemplary resonator.

In the standard configuration of the resonator, each subsequent resonator element generates a magnetic field in opposite direction. Figure S1a shows the magnetic resonator field distribution $\vec{B}_1 = B_1(x)\hat{y}$ for an ideal model resonator and a real prototype resonator with 32 active elements each. If we denote a resonator section where B_1 always points in the same direction as *stage*, we have a resonator configuration consisting of 32 stages. These numbers correspond to a configuration with $N = 16$

periods.

The ideal model is characterized by a half period $a = 1\,\text{cm}$, an infinitely thin coil thickness and vanishing distance between adjacent resonator elements. The prototype parameters are given by an inner coil distance of 1 cm as well, but now the thickness of the coil material is 0.3 mm and the distance between elements 1.0 mm, resulting in a half period $a = 1.16\,\text{cm}$.

The resonance wavelength as given by equation (2) in the main article is set to 2.6 Å. This requires a selector field $B_0 = 2.609\,\text{mT}$ in the ideal case and 2.249 mT for the prototype. An optimization procedure however yields an optimized selector field $B_0 = 2.247\,\text{mT}$. The corresponding calculations were carried out with 32 active resonator elements. According to the amplitude condition (3), the resonator field has to be set to $B_1 = 0.128\,\text{mT}$ for the ideal model. For the prototype equation (3) would yield $B_1 = 0.110\,\text{mT}$ which assumes a constant magnetic field B_1 over the complete length a. In a real configuration however, the magnetic field in the coil material and over the distance between the coils is different and also depends on the number of neighboring coils. A further optimization procedure then yields a magnetic field $B_1^{center} = 0.1160\,\text{mT}$ for $B_0 = 2.247\,\text{mT}$. Since B_1 is not constant in the real prototype the given value corresponds to the magnetic field in the center of the resonator.

Assuming an initial polarization $\vec{P} = \hat{z}$ along the $\hat{z}$-axis we may calculate the polarization of a neutron beam at the exit of the resonator, taking into account the magnetic field distributions for $B_1\hat{y}$ as given in Fig. S1a plus the constant selector field $B_0\hat{z}$. The results are shown in Fig. S1b and illustrate the $\sin^2 x/x^2$ spinflip probability as it is expressed in equation (1) of the main article.

At this point, it has to be stressed that the results shown in Fig. S1b were obtained by calculating the evolution of neutron polarization in a given configuration of a spatially varying magnetic field by a employing the dedicated software SPARTAN which was developed for exactly this purpose[4,5]. The correspondence to equation (1) is then a result of the calculation.

As can be seen from Fig. S1b the maximum spinflip probability occurs at a wavelength of 2.6 Å and its odd multiples. While for the ideal model resonator the spinflip probability for 7.8 Å neutrons is still perfect the spinflip probability for the real prototype also exhibits a local maximum at this wavelength albeit with a reduced value.

The evolution of the polarization vector throughout the real prototype resonator is illustrated in Figs. S1c and S1d for 2.6 Å neutrons. The behavior of all of its 3 components is displayed in the case of resonance. Figure S1c shows the results for a coarsely tuned resonator with $B_0^{detuned} = 2.250\,\text{mT}$ and $B_1^{detuned} = 0.1154\,\text{mT}$ in the center of the resonator. The exit polarization is characterized by a z-component P_z = -0.9968 while P_x and P_y oscillate sinusoidally about the selector field with an amplitude of about 0.07. This amplitude is a measure for the quality of resonator tuning. In an ideal case the oscillation should vanish after exit from the resonator and the polarization be aligned along the $-\hat{z}$ axis. Fine-tuning the resonator to $B_0^{tuned} = 2.247\,\text{mT}$ and $B_1^{tuned} = 0.1160\,\text{mT}$ in the center of the resonator results in P_z = -0.99999 and an oscillation amplitude of P_x and P_y of 0.0036 as illustrated in Fig. S1d. These magnetic field values clearly demonstrate how critically a high-performance resonator behavior depends on an optimized set of parameters which have to be correctly controlled and stabilized during resonator operation.

In the resonance case the polarization vector performs a single complete rotation about the selector field when traversing a full resonator period as it is demonstrated by the oscillation of P_x and P_y in Fig. S1d. The decrease of the P_z value is non-linear at the beginning and the end of the resonator while it exhibits a practically linear dependance in the center of the resonator. It follows that different resonator elements contribute differently to the change of the neutron polarization. As one could expect from a resonance configuration, the resonator has to be considered as a whole to assess its effect on the neutron polarization at a specific wavelength. Also characteristic of the real prototype resonator is the stepwise decrease of P_z which flattens in the space between adjacent resonator elements.

S2 Combining neighboring resonator elements

As discussed in the *Results* section of the main article and shown in Fig. as the results of our measurements, multiple adjacent resonator elements may be combined by having their resonator field B_1 pointing in the same direction, thereby creating a single resonator stage out of multiple resonator elements. In such a situation, the resonator itself is the same as in the previous section. Only the direction of the current generating B_1 is reversed for some of the elements. This section will demonstrate that a resonator under these conditions can again be considered as a resonator in standard configuration, although with a different number of stages, even with a realistic magnetic field distribution. Compared to the resonator in standard configuration as discussed in the previous section, the number of stages will be reduced by a factor n and the effective length of a resonator stage correspondingly increased. As a consequence, the magnetic selector field B_0 has to be decreased by the same factor n. As another consequence, the width of the central peak of $W_{\uparrow\downarrow}(\lambda)$ will be increased by this factor and the resolution changes accordingly. An increased peak width is not unwanted in all cases, as long as the requirements for the resolution are met, because it ensures a higher intensity of resonant neutrons. Such conditions may be either realized by a resonator with single-element stages with shorter overall length, (it can still be the same resonator but not all elements are switched on), or by a resonator with multi-element stages as above. Both options were realized in the experiments shown in Fig. . The advantage of the first option is shorter total length of the component, the advantage of the second option lies in the need of smaller magnetic field

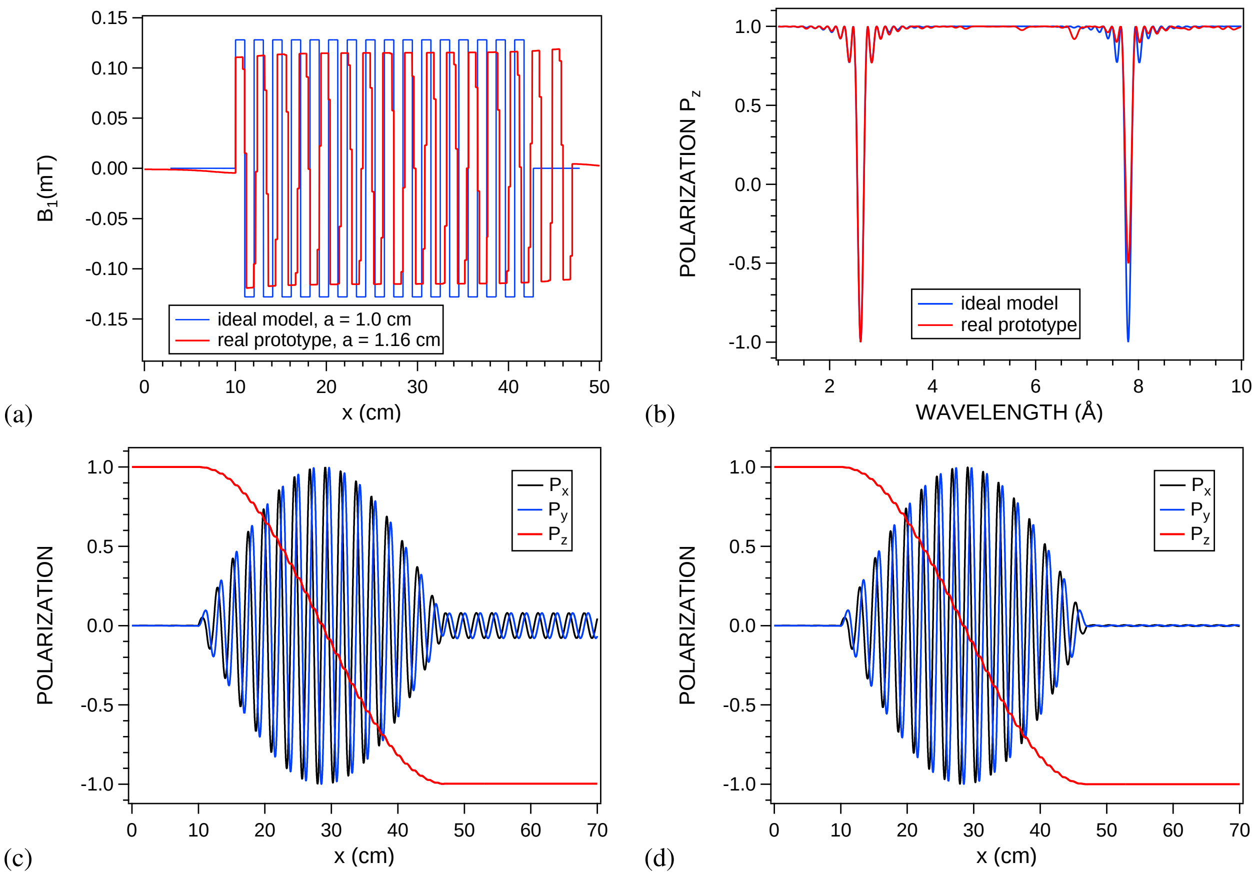

Figure S1. (a) Magnetic field configurations for 32 active elements, 32 stages and 16 resonator periods. The blue field distribution represent an ideal model, the red field distribution a real prototype resonator with finite coil thickness and distance between the resonator elements. Both magnetic field distributions are optimized for neutrons with resonance wavelength $\lambda_0 = 2.6\,\text{Å}$. (b) Spectral dependence of the polarization, expressed by its vertical component P_z, at the exit of either of the resonators with magnetic field distributions shown in (a). Wherever only the red curve is visible it lies on top of the blue one. A polarization $P_z = 1$ of the incident neutron beam is assumed. Maximum spinflip probability occurs at the resonance wavelength $\lambda_0 = 2.6\,\text{Å}$ and its odd multiples. In our specific case the first higher order maximum appears at $\lambda_1 = 3\lambda_0 = 7.8\,\text{Å}$. (c) Evolution of the polarization of 2.6 Å neutrons for a coarsely tuned real resonator with $B_0 = 2.250\,\text{mT}$, and $B_1^{center} = 0.1104\,\text{mT}$ in the center of the resonator. (d) Evolution of the polarization of 2.6 Å neutrons for a fine-tuned real resonator with $B_0 = 2.247\,\text{mT}$, and $B_1^{center} = 0.1111\,\text{mT}$ in the center of the resonator.

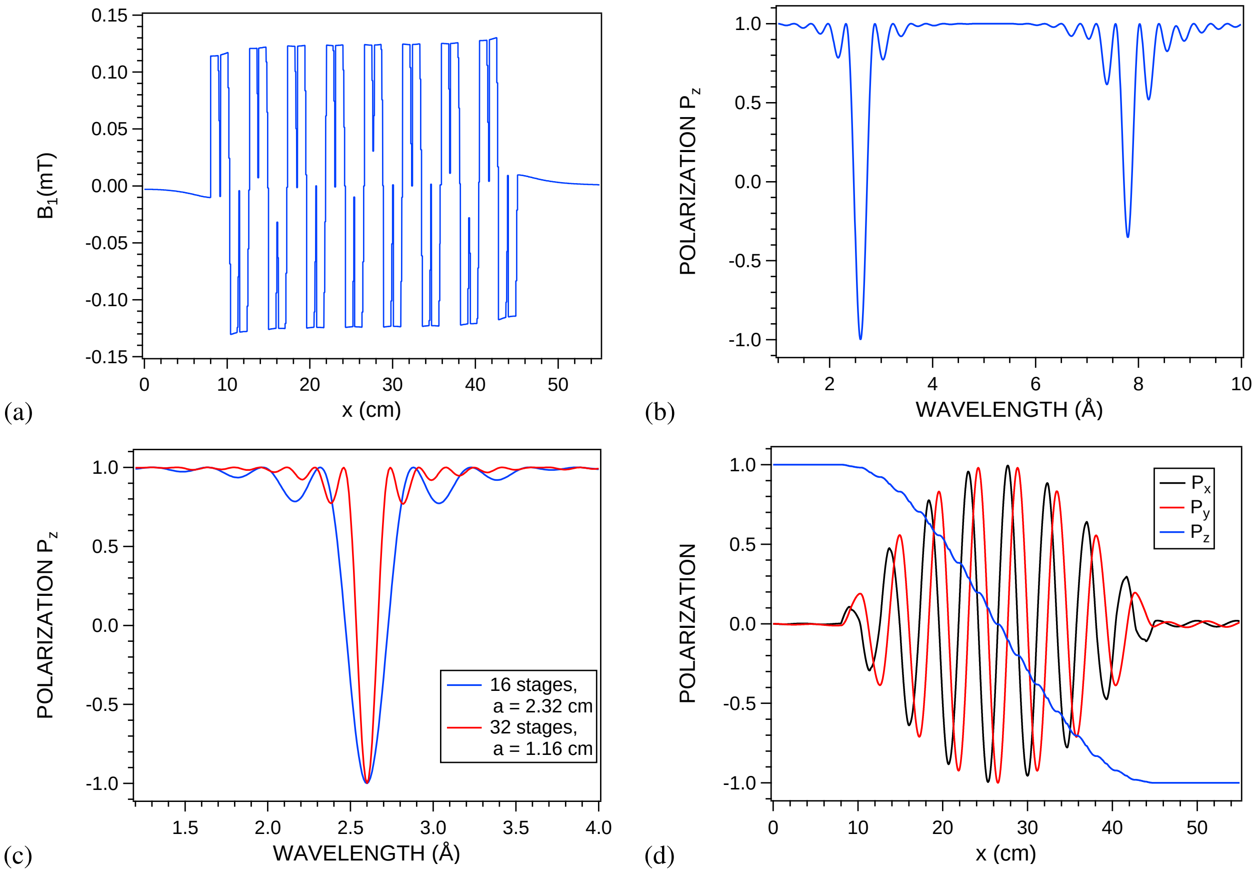

Figure S2. (a) Magnetic field configuration for the real prototype where the resonator field B_1 of 2 neighboring elements is orientated in the same direction. This configuration consists of 32 active elements, 16 stages and 8 resonator periods. The field distribution is optimized for neutrons with resonance wavelength $\lambda_0 = 2.6$ Å. (b) Spectral dependence of P_z at the exit of a resonators with the magnetic field distribution shown in (a). A polarization $P_z = 1$ of the incident neutron beam is assumed. Maximum spinflip probability occurs at the resonance wavelength $\lambda_0 = 2.6$ Å and its odd multiples, in our case at $\lambda_1 = 3\lambda_0 = 7.8$ Å. (c) Comparison of the wavelength resolution for the resonator operated with single-element (red curve) and double-element stages (blue curve). The widths of the resonances at $P_z = 0$ are ~ 0.26 Å for the resonator with 16 stages and ~ 0.13 Å for the resonator with 32 stages. (d) Evolution of the polarization for 2.6 Å neutrons passing through a 16-stage resonator under resonance conditions.

strengths, both B_0 and B_1, due to the larger effective length of a single stage, in comparison with the first option. As mentioned in the main article we had to make use of that advantage in the course of our experiments. In this section we will now illustrate the properties of the resonator introduced in the previous section when configured with multi-element stages and compare the two configurations.

In what follows we discuss the situation where 2 adjacent resonator elements are combined to form a common stage. As mentioned before, the calculations are based on the real prototype resonator of the previous section. What we then consider is a resonator configuration with 16 effective stages or $N = 8$ periods. Since the resonator elements and their respective distances are the same as before we have now a half period $a = 2.32$ cm. The magnetic field distribution $B_1(x)$ is shown in Fig. S2a. As already mentioned above, the change from the configuration as seen in Fig. S1a to the present configuration is simply achieved by inverting the direction of the current for the affected resonator coils. We find from equation (2) in the main article that by doubling the value of a we have to reduce the strength of the selector field B_0 by a factor of 2 for the same resonance wavelength λ_0. Since the number of resonator periods is reduced by the same factor, and following equation (3) of the main article, we find that the resonator field B_1 will have essentially to stay at the same value as before in order to fulfill the amplitude condition. This is intuitively readily understood in considering that at resonance the polarization vector has to make a full precession about the selector field in one resonator period which is now twice as long as before. The amplitude condition links the resonator field

B_1 to the overall length L of the resonator which in the case discussed here remains constant,

$$B_1 = \frac{\pi C}{2L\lambda_0}\,. \tag{S1}$$

An optimized set of magnetic field values for the 16-stage case consists of $B_0^{16} = 1.124\,\mathrm{mT}$, and $B_1^{16} = 0.1129\,\mathrm{mT}$ in the center of the resonator. Let us add the corresponding values for the 32-stage case which we found above, $B_0^{32} = 2.247\,\mathrm{mT}$ and $B_1^{32} = 0.1160\,\mathrm{mT}$, for immediate comparison. Indeed, we find $B_0^{32} \simeq 2B_0^{16}$ and $B_1^{32} \sim B_1^{16}$.

Assuming a perfectly polarized neutron beam with incident polarization $P_z = 1$, we calculated the polarization at the resonator exit, analogous to the results obtained for Fig. S1b. The vertical component of the polarization P_z as a function of neutron wavelength is shown in Fig. S2b for the 16-stage resonator. Conclusions for the spinflip probability concerning the resonance wavelength and its higher orders are basically equivalent to the 32-stage resonator. The oscillations around the resonances, arising from the $\sin^2 x/x^2$ behavior, are apparently more pronounced than in the 32-stage case. In particular, this is the case for the higher order resonance for which the resonator field had not been optimized. In addition to that, the spinflip probability is asymmertic around the resonance while it essentially symmetric around the resonance wavelength for which the setup had been optimized.

In Fig. S2c we compare the wavelength resolution for the resonator with 16 stages and $N = 8$ periods with the 32-stage resonator having $N = 16$ periods as discussed before. According to equation (4) in the main article the wavelength resolution is inversely proportional to the number of resonator periods. The full width at half maximum of the central peak of the spinflip probability $W_{\uparrow\downarrow}(\lambda)$ corresponds to the full width at $P_z = 0$ of the resonance minimum of the polarization component $P_z(\lambda)$. For $N = 8$ we obtain $(\Delta\lambda)_{1/2}/\lambda_0 \simeq 0.1$ and $(\Delta\lambda)_{1/2} \simeq 0.26\,\text{Å}$ at $\lambda_0 = 2.6\,\text{Å}$, For $N = 16$ the values are $(\Delta\lambda)_{1/2}/\lambda_0 \simeq 0.05$ and $(\Delta\lambda)_{1/2} \simeq 0.13\,\text{Å}$ at $\lambda_0 = 2.6\,\text{Å}$. These resonance widths are indeed reproduced in the curves shown in Fig. S2c.

The evolution of the polarization vector throughout the real prototype resonator with 16 stages is illustrated in Fig. S2d for 2.6 Å neutrons. The behavior of all of its 3 components is displayed in the case of resonance. This figure may be compared with Figs. S1c and S1d for the case of 32 stages. The exit polarization after the resonator is characterized by $P_z =$ -0.99982 while P_x and P_y oscillate sinusoidally about the selector field with an amplitude of about 0.018. Again, at resonance the polarization vector performs a single complete rotation about the selector field during the passage of a full resonator period. The phenomenology corresponds to the discussion related to Fig. S1d.

S3 Increasing the resolution

Following the discussion related to Fig. S2c we conclude that we may improve the wavelength resolution of the resonator by decreasing the value for the half period a while keeping the overall length of the resonator constant. The same would hold true if we keep the value of a constant and increase the total length of the resonator. Both measures effectively result in an increase of the number of resonator periods N. This is exactly what is expressed by equation (4) in the main article. For a given resonator this number is of course limited by the number of available resonator elements. A different option to increase the wavelength resolution follows the discussion related to equation (5) in the main article. This option takes advantage of the higher order resonances, i.e. resonances that are odd multiples of the resonance wavelength λ_0. In this section we will illustrate the properties of higher order resonances by the spectral behavior of the spinflip probability as well as by the evolution of the neutron polarization, where we find an increased complexity with increasing order number. Although the related experiments presented in the main article were performed with multi-element stages we will limit this section to the employment of single-element stages to highlight the effect of higher order resonances without any further complexity.

Figure S3a illustrates the situation for a 25 stage resonator with a half period $a = 2.16\,\mathrm{cm}$. This configuration has been motivated in two ways. First, we wanted to have a resonator with a smaller number of resonator stages than the one presented in section S1, which renders the plots showing the evolution of the neutron polarization better readable. Second, with the choice of an odd number of stages we are able to demonstrate that a perfectly working resonator is not limited to an even number of stages, even in the more complex case of a higher order resonance.

Optimized resonance conditions for this resonator and a resonance wavelength of 2.63 Å yield $B_0 = 1.192\,\mathrm{mT}$, and $B_1^{center} = 76.65\,\mu\mathrm{T}$ in the center of the resonator. As discussed in the context of Fig. S1b these parameters are, however, not perfectly suitable for the higher order resonances. While the exit polarization component P_z is -0.99981 for $\lambda_0 = 2.63\,\text{Å}$ neutrons, its value amounts to -0.9229 for $\lambda_1 = 7.89\,\text{Å}$ neutrons and to only -0.5352 for $\lambda_2 = 13.15\,\text{Å}$ neutrons.

The evolution of the polarization vector throughout the real prototype resonator with 25 stages is illustrated in Fig. S3b for 2.63 Å neutrons. The behavior of all of its 3 components is displayed in the case of resonance which is defined by the parameters given in the preceding paragraph. This figure may be compared with Fig. S1d for the case of 32 stages and Fig. S2d for the case of 16 stages. The exit polarization after the resonator is characterized by $P_z =$ -0.99981 as already mentioned above while P_x and P_y oscillate sinusoidally about the selector field with an amplitude of about 0.020. A qualitative difference to the

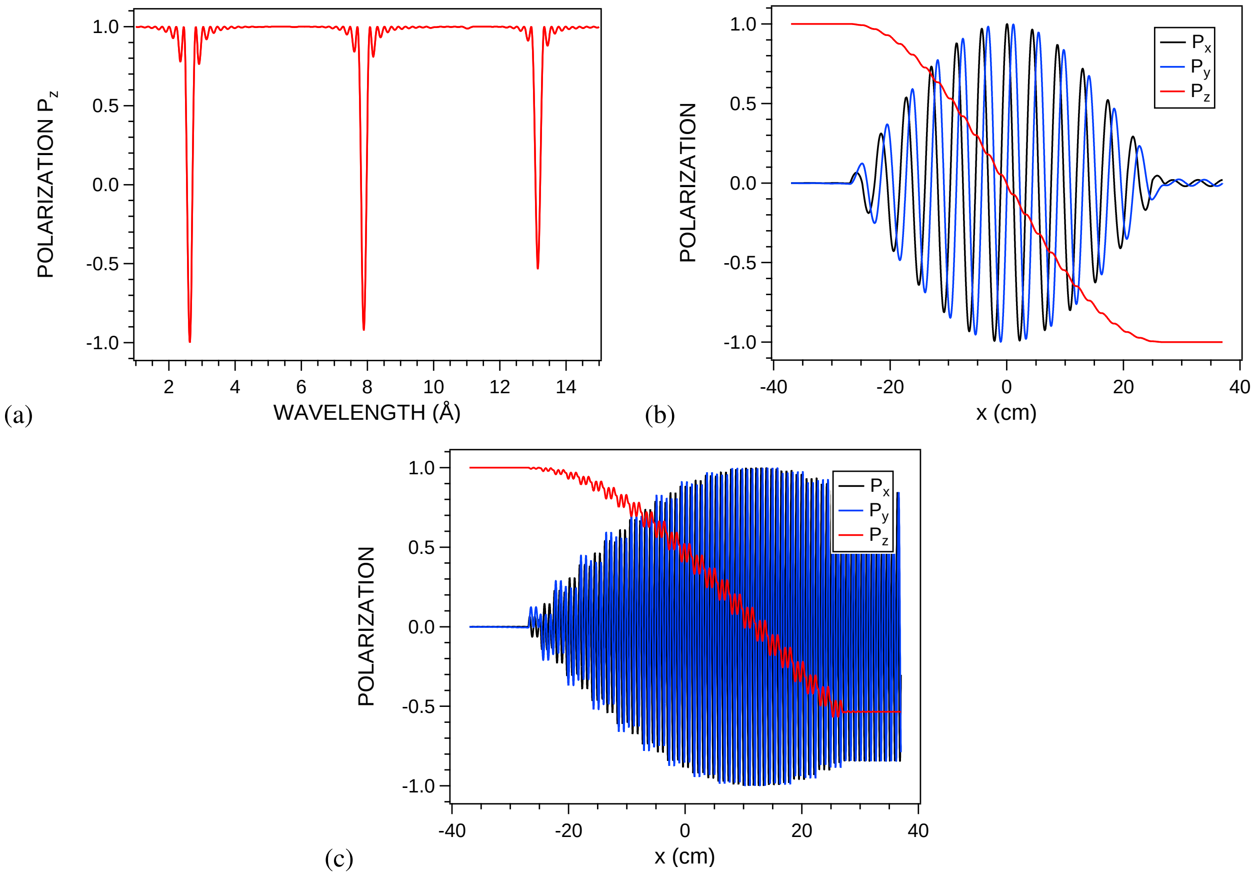

Figure S3. (a) Exit polarization P_z as a function of neutron wavelength for a resonator with 25 stages and the setup optimized for $\lambda_0 = 2.63$ Å neutrons, $B_0 = 1.192$ mT, $B_1^{center} = 76.65$ µT. The higher order resonances are clearly seen at $\lambda_1 = 7.89$ Å and $\lambda_2 = 13.15$ Å, however their heights do not reach their optimal values because of improper field parameters (see text). (b) Evolution of the polarization for 2.63 Å neutrons passing through a 25-stage resonator under resonance conditions for this wavelength. (c) Evolution of the polarization for $\lambda_2 = 13.15$ Å neutrons throughout a resonator optimized for 2.63 Å.

two cases discussed before arises from the fact that the number of resonator elements is an odd number, he 25-stage resonator consists of 12 1/2 periods. The polarization vector of the resonant neutrons performs 13 full rotations about the selector field, although the last half rotation with a very small amplitude of P_x and P_y. There the polarization vector is practically aligned along the $\hat{z}$-axis and characterized by $P_z \sim -1$.

The situation as discussed before may be compared to the evolution of the polarization vector for 13.15 Å neutrons throughout the real prototype resonator with 25 stages with the same parameters as before. This is shown in Fig. S3c. As was already seen in Fig. S3a the exit polarization for this wavelength is characterized by $P_z =$ -0.5352 while the horizontal components of the polarization vector continue to oscillate with an amplitude of 0.84 about the selector field. In this case the resonator field B_1 is not sufficiently large enough to allow for a complete inversion of the initial polarization. Since the selector field is 5 times larger than we would choose it for $\lambda_0 = 13.15$ Å the polarization vector performs approximately 5 rotations per resonator period instead of the one shown in Fig. S3b.

However, the resonator parameters may be optimized to yield an improved spinflip probability at 13.15 Å, although at the cost of a reduced performance for neutrons with the resonance wavelength λ_0. From the previous discussion we can expect an increase of B_1 and a marginally changed value of B_0 to accommodate for the special situation. Indeed, an optimization procedure finds, $B_0 = 1.1894$ mT and $B_1^{center} = 111.85$ µT. The new value for B_0 is 99.8% of the original one while B_1 is at 146% of the original value. This considerable increase of B_1 is a direct consequence of the real prototype resonator while the values would remain unchanged for an ideal resonator configuration. With the new set of parameters the exit polarization for 2.63 Å neutrons is now characterized by $P_z =$ -0.0983, and for 7.89 Å neutrons by $P_z =$ -0.6430 while at 13.15 Å we obtain $P_z =$ -0.9994. These results are summarized by showing P_z as a function of neutron wavelength for the modified parameter set

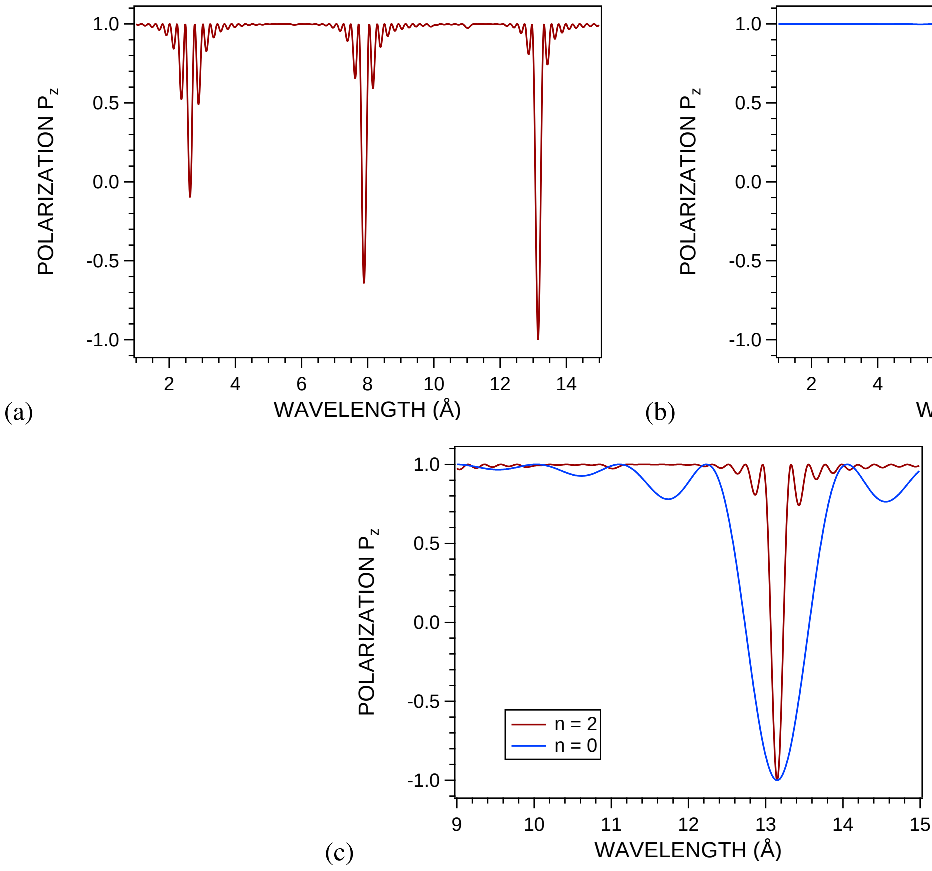

Figure S4. (a) Exit polarization P_z as a function of neutron wavelength of the setup with modified parameters, $B_0 = 1.1894\,\mathrm{mT}$, $B_1^{center} = 111.85\,\mu\mathrm{T}$. These settings define an $n = 2$ condition for 13.15 Å neutrons. (b) Exit polarization P_z as a function of neutron wavelength of the setup with parameters optimized for 13.15 Å neutrons, $B_0 = 0.2384\,\mathrm{mT}$, $B_1^{center} = 15.24\,\mu\mathrm{T}$. These settings define an $n = 0$ condition. (c) Comparison of the resonance widths for 13.15 Å neutrons with the resonator operated in $n = 0$ and $n = 2$ conditions.

in Fig. S4a.

In the previous discussion we have started with a resonance wavelength $\lambda_0 = 2.63\,\text{Å}$ and optimized the resonator parameters for the higher order wavlength $\lambda_2 = 5\lambda_0 = 13.15\,\text{Å}$. If we now choose the latter wavelength as resonance wavelength we have to optimize the resonator parameter for $\lambda_0' = 13.15\,\text{Å}$. From equations (2) and (3) of the main article we should expect to find these parameters by reducing the magnetic field values for 2.63 Å neutron by a factor $\lambda_2/\lambda_0 = 5$. An optimization procedure yields $B_0 = 0.2384\,\mathrm{mT}$ and $B_1^{center} = 15.24\,\mu\mathrm{T}$ for 13.15 Å neutrons. These values for B_0 and B_1 correspond indeed to one fifth of the values found for 2.63 Å neutrons. The related exit polarization at the end of the resonator is visualized by the component P_z as a function of neutron wavelength in Fig. S4b. This figure may be compared with the previous figure S4a which are both plotted for the same wavelength range. Two features become immediately apparent. Since λ_0' is the resonance wavelength there are no additional resonances visible at shorter wavelengths in Fig. S4b. Second, the resonance at 13.15 Å appears much broader in Fig. S4b than in Fig. S4a.

Figure S4c compares the two resonances by plotting them into the same picture. Following the discussion in the *Results* section of the main article we identify the settings for λ_2 as an $n = 2$ configuration and the settings for λ_0' as an $n = 0$ configuration. According to equation (5) the resonance width should be reduced a factor $2n+1$ in the case of a higher order resonance. Indeed, we find that the $n = 2$ resonance width, $\Delta\lambda_{1/2}(2) = 0.1688\,\text{Å}$, is 5 times smaller than the width for $n = 0$, $\Delta\lambda_{1/2}(0) = 0.84\,\text{Å}$.

Let us finally compare the evolution of the polarization vector throughout the resonator for the various configurations related to resonance wavelength $\lambda_0 = 2.63\,\text{Å}$. For settings optimized for this wavelength the evolution of the polarization was already shown in Fig. S3b. For the $n = 2$ higher order resonance discussed before an equivalent picture is shown in Fig. S5a.

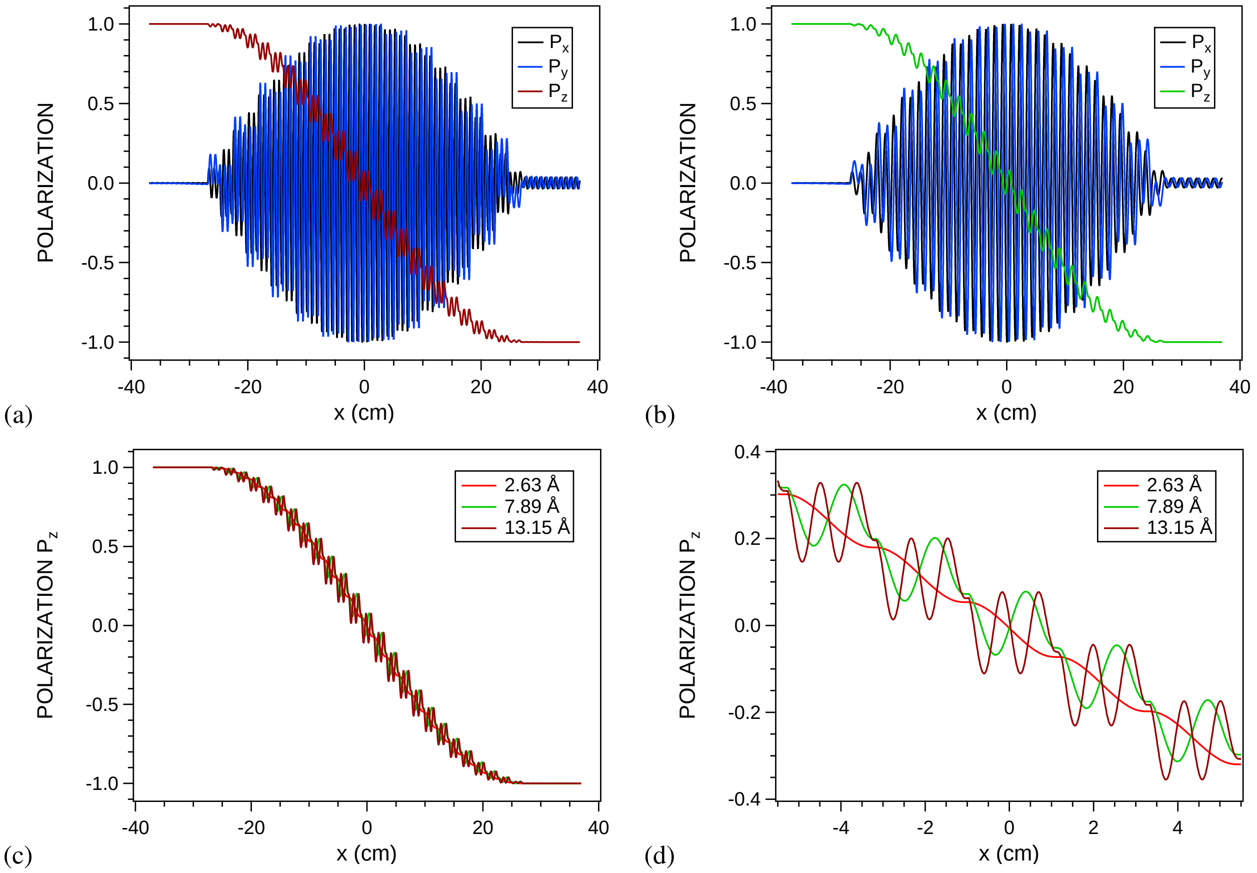

Figure S5. (a) Evolution of the polarization for 13.15 Å neutrons with the modified setup as shown in Fig. S4a, $B_0 = 1.1894\,\mathrm{mT}$, $B_1^{center} = 111.85\,\mu\mathrm{T}$. (b) Evolution of the polarization for 7.89 Å neutrons with an again modified 2.63 Å setup tailored to optimum conditions for the 7.89 Å neutrons, $B_0 = 1.191\,\mathrm{mT}$, $B_1^{center} = 86.90\,\mu\mathrm{T}$. (c) Comparison of the evolution of the polarization component P_z for 2.63 Å neutrons, 7.89 Å neutrons, and 13.15 Å. In all 3 cases $\lambda_0 = 2.63\,\text{Å}$ is selected but the parameters are optimized for the respective wavelength in each case. (d) Detailed view of (c) in the center of the resonator.

Here, the parameters have been optimized with respect to $\lambda_2 = 13.15\,\text{Å}$ in contrast to Fig. S3c. Eventually, the evolution of the neutron polarization with parameters optimized for the $n = 1$ higher order resonance at $\lambda_1 = 7.89\,\text{Å}$ is shown in Fig. S5b. Both graphs, Figs. S5a and S5b, exhibit an increased number of rotations about the vertical selector field B_0 when compared to the case of the resonance wavelength as shown in Fig. S3b. In all cases the optimization procedure ensures that the magnitude of the components P_x and P_y is minimized after traversing the resonator and an almost perfect inversion of the initial polarization takes place.

The evolution of the polarization component P_z for the 3 cases is compared in Figs. S5c and S5d. Starting with $P_z = 1$ at the beginning of the resonator, the exit polarization reaches values of P_z better than 0.999 for each configuration. Throughout the resonator the values for P_z follow a more or less complicated pattern with increasing complexity for larger values of n. A detailed picture of that situation is given in Fig. S5d which covers the range of about 5 stages or 2 1/2 resonator periods. For the resonance wavelength λ_0 $P_z(x)$ is a monotonically decreasing function from the entry into the resonator until the exit. The decrease is steeper within the resonator coils and flattens in the regions between the coils. For higher order resonance wavelengths $P_z(x)$ is still an effectively decreasing function but exhibits, as the horizontal components of the polarization vector, an oscillatory behavior. Thereby, the number of oscillations within one stage is given by the order number n.

References

1. CST Computer Simulation Technology AG. CST® Studio Suite® 2012. http://www.cst.com/.
2. Gösselsberger, C. *Entwicklung eines Wanderwellen-Neutronenspinresonators*. Ph.D. thesis, TU Wien (2012).

3. Drabkin, G. M., Trunov, V. A. & Runov, V. B. Static Magnetic Field Analysis of a Polarized Neutron Spectrum. *Sov. Phys. JETP* **27**, 194–196 (1968).

4. Welzl, A. SPARTAN: Software zur Simulation des Neutronen-Spin-Flips. Bachelor thesis, TU Wien (2010).

5. Welzl, A. SPARTAN II: Software zur Simulation des Neutronen-Spin-Flips. Project thesis, TU Wien (2011).
